# Radiocarbon dating of prehistoric phytoliths: a preliminary study of archaeological sites in China

**DOI:** 10.1038/srep26769

**Published:** 2016-05-26

**Authors:** Xinxin Zuo, Houyuan Lu, Jianping Zhang, Can Wang, Guoping Sun, Yunfei Zheng

**Affiliations:** 1 Key Laboratory of Cenozoic Geology and Environment, Institute of Geology and Geophysics, Chinese Academy of Sciences, 100029, Beijing, China; 2 Center for Excellence in Tibetan Plateau Earth Science, Chinese Academy of Sciences, 100101, Beijing, China; 3 Zhejiang Provincial Institute of relics and Archaeology, Jiashan Road, 310014, Hangzhou, China

## Abstract

Phytoliths can occlude some organic carbon during their deposition in plants. This carbon fraction is recognised as an ideal dating material because of its high resistance to decomposition and post-deposition contamination at the time of phytolith formation. However, the reliability of phytolith radiocarbon dating has recently been questioned. The development of a new extraction protocol for phytoliths, with paired dating between phytoliths and other materials from the same sediment, may provide further evidence for the reliability of phytolith dating. We present an improved method for extracting phytoliths from soils. We compared the dating of phytoliths and other materials (e.g., charcoal and plant seeds) recovered at the same depth from seven pits at six archaeological sites in China. The estimated ages of the phytoliths and other materials were generally consistent, except for one outlier. We attribute this inconsistency to the post-depositional processes of phytoliths in soil, rather than to the uptake of old carbon from the soil. Our results clearly show the potential for phytolith carbon dating at archaeological sites in the absence of other dating materials.

Radiocarbon dating has proven to be a powerful tool for reliably obtaining the ages of past events recorded in sediments and archaeological sites during the late Quaternary period. However, the selection of materials has a profound effect on the quality of radiocarbon dating^1^. Wood, plant residue, and charcoal are generally accepted as robust dating materials because of their homogeneity and relatively good preservation^2^. However, these remains are often absent from many sedimentary archives and archaeological sites. Consequently, it is necessary to identify alternative materials that might enable reliable and effective dating.

Phytoliths(SiO_2_·nH_2_O) are non-crystalline minerals that are deposited within the cells and cell walls in various parts of plants^3^. Some organic carbon of plant origin is occluded by phytoliths during their deposition^4^,^5^. When the plant dies and decays, this carbon fraction, encapsulated by silica, can survive for long periods due to the phytolith’s high resistance to decomposition. Phytolith-occluded carbon (PhytOC) has been demonstrated to be an important form of carbon sequestration^6^,^7^,^8^,^9^. Because PhytOC is usually taken to be a product of photosynthesis, it has been used to reconstruct C3/C4 plants of the past^10^,^11^,^12^, for paleo-CO_2_ concentration^13^, and in radiocarbon dating tests^4^,^14^,^15^,^16^,^17^,^18^.

The earliest radiocarbon dating studies using phytoliths were carried out by Jones and Beavers^19^ and Wilding *et al.*^14^. They investigated the potential of PhytOC for radiocarbon analysis, and found that the measurements obtained using phytoliths were older than those expected sediments developed from the soil. Studies on phytolith dating of lake, terrestrial soil sediments, and archaeological sites showed good agreement between phytolith dating and methods utilizing other dating materials^4^,^16^,^17^,^20^,^21^,^22^,^23^. However, in a small number of studies, phytolith dating was attempted but was unsuccessful because no expected phytoliths ages were retrieved^1^,^24^. A few studies attributed this distortion of phytolith dating to old carbon absorbed from soils^25^,^26^,^27^. Further testing of phytolith dating at archaeological sites is required to confirm whether or not phytolith dating can be influenced by the carbon content of old soils.

In this study, we collected palaeosoil samples from pits dug at archaeological sites in China. A new, improved method was developed to extract phytoliths from soils. Scanning electron microscopy–energy-dispersive X-ray spectroscopy (SEM-EDS) analysis was performed to check the purity of concentrated phytoliths. Then, the pure phytolith and other dating materials were dated by accelerator mass spectrometry. Finally, phytolith dating was compared with dating results obtained using other materials (charcoal, plant residue) recovered from the same pit depth or cultural layer.

## Materials and Method

**Figure 1:**
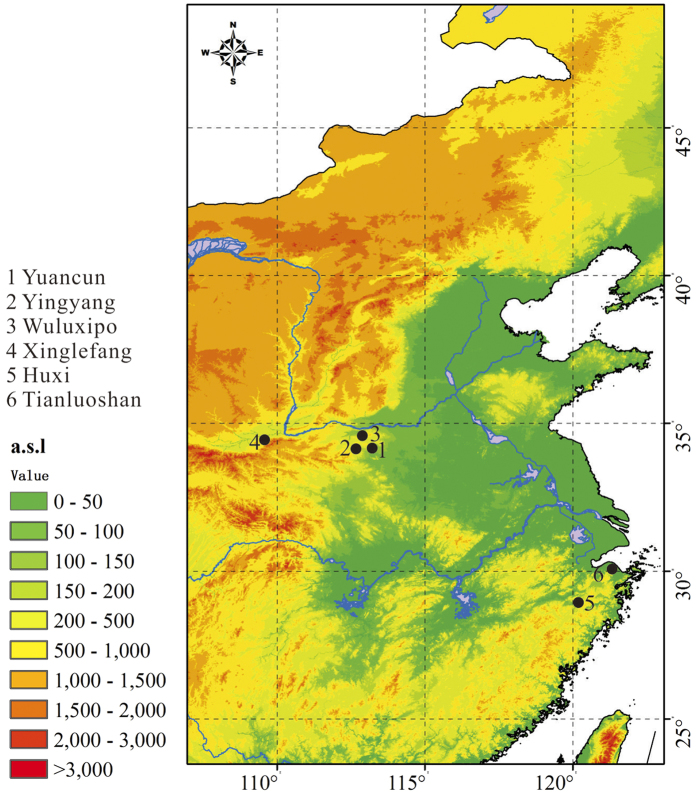
Locations of archaeological sites The figure was generated using GRASS GIS 7.0.3: https://grass.osgeo.org/.

Fourteen samples were collected from six archaeological sites. Soil and charcoals or seeds were simultaneously selected at the same depth from the pits and cultural layers. The Tianluoshan and Huxi sites are located in Zhejiang province, southeastern China. The Yingyang, Yuancun, and Wuluoxipo sites are located in Henan province, central China. The Xinglefang site is located in Shanxi province, western China (Fig. 1). Wuluoxipo is attributed to the Peiligang culture (7000–5000 BCE). Yingyang and Yuancun are attributed to the Yangshao culture (5000–3000 BCE). Xinglefang is attributed to the Miaodigou culture (3900–3600 BCE). Huxi and Tianluoshan are attributed to the Shangshan (8000–5500 BCE) and Hemudu (5000–4000 BCE) cultures, respectively (Table 1)^28^.

A modified wet oxidation method was used for extracting phytoliths from soil^3^,^13^,^29^,^30^. The detailed steps are as follows: (a) Dry soil was crushed and sieved at 500 μm; (b) The sample was deflocculated with 5% sodium polyphosphates, and then washed three to four times with distilled water; (c) Organic matter was first oxidised by 250 ml of H_2_O_2_ (30%) for 12 h at room temperature and then heated in a water bath until the reaction stopped; (d) Carbonates were eliminated using 200 ml of HCl (10%) with heating for 30 min; (e) The >250 μm fraction was separated by wet sieving, and the remaining sample was disaggregated from the organic material and clay by ultrasonic treatment for 20 min; (f) Clays (<5 μm) were removed by gravity sedimentation until the sample was clear; (g) The remaining higher-resistance materials were oxidised by 200 ml of HNO_3_ and pinches of KClO_3_ with heating for 1 h, and were then centrifuged and decanted; (h) Phytoliths were extracted three times by 200 ml of heavy liquid (ZnBr2) with a specific density of 2.35 g/cm^3^ and then washed three times with distilled water; (i) Extracted phytoliths were further sieved at 7 μm to remove clay. Then, the recovered part of remains in the sieve were treated by 20 ml of H_2_O_2_ (30%) in the tube for 20 min; (j) Finally, the recovered phytoliths were dried at 60 °C for 24 h prior to testing.

The phytolith and most of the other materials were dated by Beta Analytic Lab, except for two plant samples from the Tianluoshan site, which were sent to the Peking University accelerator mass spectrometry (AMS) laboratory. The phytolith dating processes can generally be described by the following three steps: First, the sample is placed into a combustion vessel (quartz glass) and combusted at 1500 °C to generate CO_2_. The high temperature is necessary to melt the phytolith and ensure that all the carbon is combusted. Secondly, the CO_2_ is collected and converted to graphite. Finally, the graphite is measured by the accelerator mass spectrometer.

The purity of the phytoliths was checked by SEM-EDS analysis. This is recognised as a robust method for checking phytolith purity and has also been applied to evaluating routine extraction processes^26^,^31^. The steps were previously described by Corbineau *et al.*^31^. In this study, the extracted phytoliths were analysed using an SEM (LEO1450VP) in association with an EDS system (INCA ENERGY 300).

## Results

**Figure 2:**
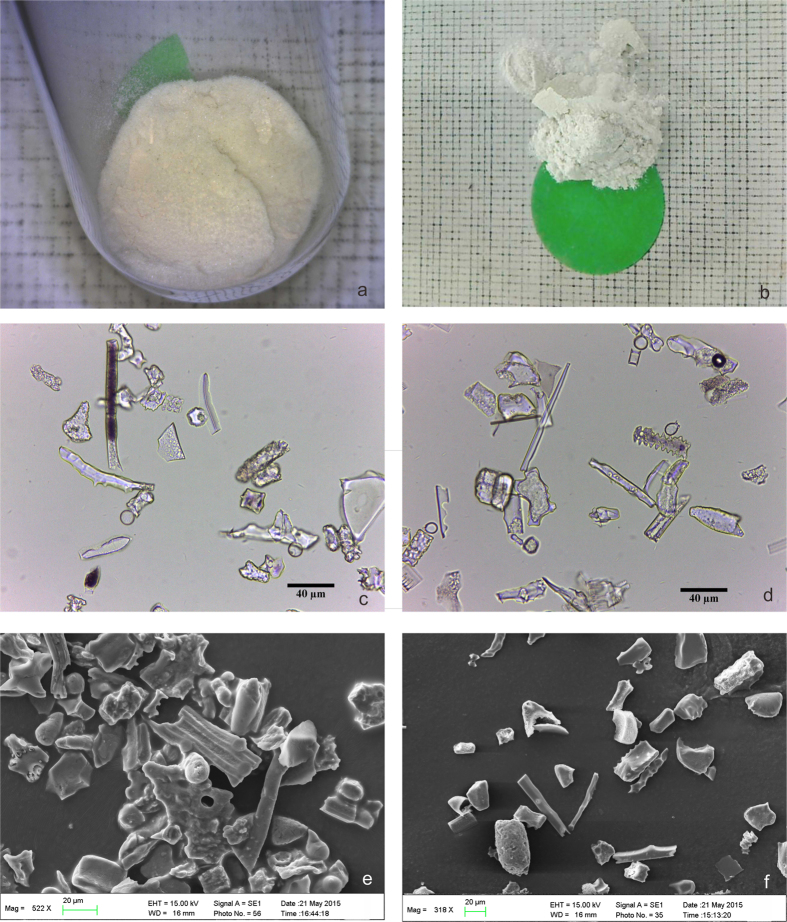
Images of phytoliths extracted from soils: (**a,b**) images of phytoliths; (**c,d**) optical microscopy images of phytoliths; (**e,f**) SEM images of phytoliths.

As shown in Fig. 2a,b, the extracted phytoliths appeared as white or grey-white. None of the charcoal or clays were observed with an optical microscope (Fig. 2c,d). The absence of extraneous organic materials was further checked by SEM-EDS analysis (Fig. 2e,f). Four micro-areas on the phytoliths were randomly selected for EDS analysis. The EDS spectrum showed two peaks caused by X-rays that were given off as electrons returning to the Si and O electron shells. The Si and O comprised more than 90% of the total mass, and the atomic ratio was nearly 2:1. Note that a few K atoms were detected in the EDS analysis of a micro-area; however, no C was found in the EDS spectra of the phytoliths.

All conventional ages were calibrated to calendar years using Calib Rev 7.0.4 and the IntCal13 calibration curve^32^. The ages of the phytoliths were consistent with the cultural periods. Thus, all of the dating results were generally acceptable, regardless of which materials were being dated. In general, the phytolith dating results were concordant or similar to those of other materials, except for one sample from the YY site, which indicated an age 1000 years older than the charcoal date. The results listed in Table 2 can be categorised into three groups: (1) phytolith dating substantially consistent with the other materials within an error bar of 2σ (HX, WLXP, and TLS-3); (2) phytolith dating within ±200–300 years of dating with the other materials (XLF, YC, and TLS-2); and (3) phytolith dating was an outlier, and thousands of years older than the dating with other materials (YY). The detailed extracted phytoliths from soils, analysed phytoliths for combustion, graphite, and carbon yield rates are shown in Table S1.

## Discussion

The extraction of pure phytolith content is of fundamental importance to radiocarbon dating. In our previous experiments, the conventional extraction methods that only involve H_2_O_2_ and HCl pretreatment were usually unable to exclude all exogenous organic materials and clays^33^. Thus, the ages of phytoliths were likely distorted when employing the conventional extraction method^27^. In this study, we developed three stages of sieving for our extraction protocol. Firstly, plant residues and roots are sieved at 500 μm. Secondly, macro-charcoal and micro-plant residues are sieved at 250 μm. Finally, extracted phytoliths are sieved at 7 μm to remove clay. Exogenous organic materials are excluded by H_2_O_2_ and acid. Rapid digestion (H_2_SO_4_ and H_2_O_2_) has previously been used for the extraction of phytoliths^30^,^34^. A recent study argued that rapid digestion was so harsh that it led to the consumption of carbon occluded in phytoliths^35^. Hence, we used HNO_3_/KClO_3_ rather than rapid digestion. This improved method is widely employed to extract soil phytoliths for isotopic analysis^3^, and has proven to be efficient for the removal of organic materials.

**Figure 3:**
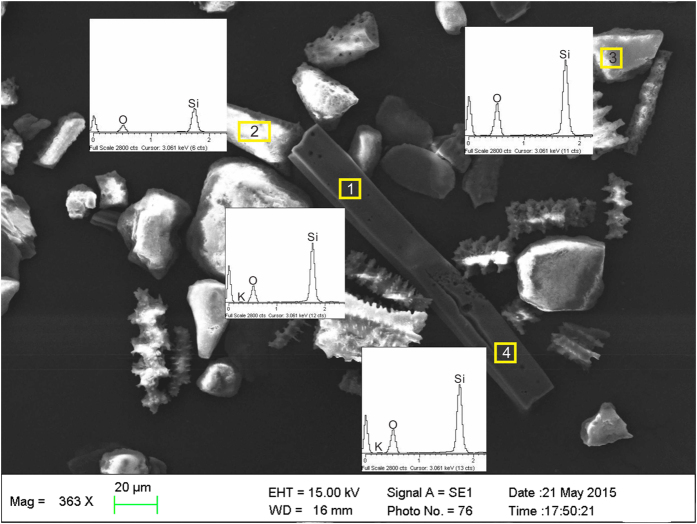
EDS analysis of phytolith surface. 1 and 4 are EDS spectra of elongate phytoliths; 2 is EDS spectrum of acicular phytolith; 3 is EDS spectrum of square phytolith.

Based on Fig. 2, we conclude that extracted phytoliths vary in colour from white to grey-white. Exogenous organic materials and clays were not detected in the microscopic examination. EDS analysis indicated that Si and O were the main elements of the phytoliths. No carbon was found in the EDS results (Fig. 3). The analysis results verified the purity of the phytoliths extracted using the improved method.

**Figure 4:**
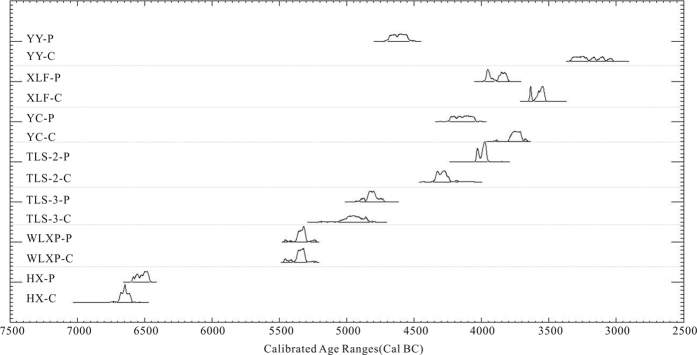
Calibrated two-sigma probability distributions for radiocarbon assays of phytoliths and other materials.

Table 2 and Fig. 4 show AMS radiocarbon dating of phytoliths and other materials. Three phytoliths dates partly or completely overlapped with the other materials within an uncertainty of 2σ, which confirmed their concordance. Three other phytolith dating results were slightly older or younger (<300 years) than those for other materials. A portion of the soil phytoliths was probably inherited from previous grasses, demonstrating a long deposition history of the soil phytoliths^29^,^36^. In this case, the soil phytolith dating results could only represent the mean yielded time of phytoliths. Due to their differing depositional processes in soils, phytoliths may have different ages from that of charcoal at the same soils profile depth^18^. When sampling a thick soil layer of 5–10 cm, a difference of hundreds of years between the dating results of soil phytoliths and other materials is generally acceptable. However, that does not account for the discrepancy of thousands of years, between the dates for one phytolith/non-phytolith pair obtained from one pit. The post-dispositional processes of phytoliths in archaeological pits might be considered for a possible explanation.

Post-depositional movements of phytoliths after depositing in pits have a fundamental effect on either the chronology or composition of phytoliths^37^,^38^. Phytoliths are subjected to translocation, bioturbation, and stratigraphic mixing processes after being incorporated into a soil^18^,^39^. Heavy translocation and extreme bioturbation may produce a phytolith pool that differs in chronology and composition even given the same soil profiles^38^. Based on the depositional processes described above, we believe that the discrepancy between the charcoal and phytolith dating results at the YY site is likely due to vertical translocation of the phytolith composition within the sequence.

In this study, the dating results of six phytolith samples were generally consistent with those of other dating materials, except for one sample from the pits of the YY site. Our results pose questions concerning the presence in phytoliths of old carbon taken up by plants from soils. Although discussion on this issue is ongoing^25^,^40^,^41^,^42^,^43^, it is probably not the most important factor for consideration, at least in prehistoric phytolith dating. Further data are required for deeper discussion on the issue of old carbon within phytoliths.

## Conclusions

In this study, we present an improved method for extracting phytoliths from pits and cultural layers. The proposed method was employed to compare dating results obtained from phytoliths with those of other common materials at the same depth, for materials recovered from seven pits or cultural layers at six archaeological sites in China. We found that phytolith carbon dating could provide a reliable and accurate chronometer. The ages of soil phytoliths were generally consistent with those of other dating materials sampled at the same depths within the pits and cultural layers. We speculate that the observed inconsistencies can be attributed to the post-depositional processes of phytoliths. Our results do not support that phytolith dating could be distorted by the presence of old carbon, absorbed by plants from soils. However, we emphasise the importance of extracting pure phytolith from soils for dating tests.

## Additional Information

**How to cite this article**: Zuo, X. *et al.* Radiocarbon dating of prehistoric phytoliths: a preliminary study of archaeological sites in China. *Sci. Rep.* **6**, 26769; doi: 10.1038/srep26769 (2016).

## Supplementary Material

Supplementary Information

## Figures and Tables

**Table 1:** Sites, locations, and weights of selected samples.

Archaeological sites	Sample code	Location	Cultural period	Other dating materials	Soil dry weight (g)
Yuancun	YC	Henan province	Yangshao	Charcoal	25.024
Yingyang	YY	Henan province	Yangshao	Charcoal	135.645
Wuluoxipo	WLXP	Henan province	Peiligang	Charcoal	137.222
Xinglefang	XLF	Shanxi province	Miaodigou	Charcoal	80.16
Huxi	HX	Zhejiang province	Shangshan	Plant residue	102.4
Tianluoshan	TLS-2	Zhejiang province	Hemudu	Seeds	70.513
Tianluoshan	TLS-3	Zhejiang province	Hemudu	Seeds	56.75

**Table 2:** AMS radiocarbon dating results with uncertainty ±2σ

Lab ID	Archaeological sites	Sample code	Dating materials	^13^/^12^C Ratio	Conventional age (BP)	2σ Calibration (Cal BC)
Beta-407469	Huxi	HX-C	Plant remains	−25.9 _0/00_	7820 ± 30	6690–6595
Beta-406654	Huxi	HX-P	Phytolith	−25.7 _0/00_	7680 ± 30	6590–6460
Beta-404827	Wuluoxipo	WLXP-C	Char	−25.5 _0/00_	6360 ± 30	5460–5450
Beta-404848	Wuluoxipo	WLXP-P	Phytolith	−26.0 _0/00_	6350 ± 30	5370–5300
BA07763	Tianluoshan	TLS-3-C	Flatstalk bulrush	NA	6045 ± 45	5060–4800
Beta-409348	Tianluoshan	TLS-3-P	Phytolith	−32.1 _0/00_	5940 ± 30	4895–4865
BA08204	Tianluoshan	TLS-2-C	Yagara bulrush seed	NA	5430 ± 40	4200–4170
Beta-409347	Tianluoshan	TLS-2-P	Phytolith	−31.2 _0/00_	5180 ± 30	4040–3955
Beta-392838	Xinglefang	XLF-C	Char	−24.9 _0/00_	4800 ± 30	3645–3625
Beta-409349	Xinglefang	XLF-P	Phytolith	NA*	5110 ± 30	3970–3910
Beta-404835	Yuancun	YC-C	Char	−25.2 _0/00_	4970 ± 30	3890–3885
Beta-404844	Yuancun	YC-P	Phytolith	−24.6 _0/00_	5310 ± 30	4240–4040
Beta-404837	Yingyang	YY-C	Char	−26.0 _0/00_	4470 ± 30	3340–3080
Beta-404846	Yingyang	YY-P	Phytolith	−24.4_0/00_	5760 ± 40	4710–4500

^*^The original sample was too small to provide a δ^13^C on the original material. However, a ratio including both natural and laboratory effects was measured during ^14^C detection in order to calculate the true Conventional Radiocarbon Age.
