# Supplementary material for: Radiocarbon dating of prehistoric phytoliths: a preliminary study of archaeological sites in China
Source: Sci Rep. 2016 May 26;6:26769. doi: 10.1038/srep26769 (PMC4880898; doi:10.1038/srep26769)
Supplement: Supplementary Information [file srep26769-s1.docx]

**Radiocarbon dating of prehistoric phytoliths: a preliminary study of archaeological sites in China**

**Xinxin Zuo^1*^, Houyuan Lu^1,2*^, Jianping Zhang^1,2^, Can Wang^1^, Guoping Sun^3^, Yunfei Zheng^3^**

^1^Key Laboratory of Cenozoic Geology and Environment, Institute of Geology and Geophysics, Chinese Academy of Sciences, 100029, Beijing, China; ^2^Center for Excellence in Tibetan Plateau Earth Science, Chinese Academy of Sciences, 100101, Beijing, China; ^3^Zhejiang Provincial Institute of relics and Archaeology, Jiashan Road, 310014, Hangzhou, China

*Corresponding author

Email: zuoxinxin@live.cn

Tel: +86 10 8299 8548

Fax: +86 10 6201 0846

Postal address: No. 19, Beitucheng Western Road, Chaoyang District, 100029, Beijing, China

This Supplementary Text file includes:

Table S1

Table S1 The extracted phytolith, finally analyzed phytolith, and detailed phytoliths dating results. Beta Lab do much better than just running procedural blanks. They run procedural standards. These are materials of known age which are treated identically to the unknown samples. The results must be within 2 sigma of the expected values for them to accept the results. Procedural blank is of course run also, but this is standard practice and used as the background to obtain the age of the both the unknowns and the procedural standards. The minimum analyzed phytolith for combustion was large than 122 mg. All the samples were large by AMS standards (> 190 ug C).

| Sample code | Beta ID | Extracted  (mg) | Analyzed  (mg) | Graphite(mg C) | Carbon yield rate(%) | Fraction Modern ± 1σ |
| --- | --- | --- | --- | --- | --- | --- |
| XLF-P | 409349 | 510 | 461.7 | 0.19 | 0.04 | 0.5293 ± 0.0020 |
| TLS-3-P | 409348 | 520 | 486.3 | 2.52 | 0.52 | 0.4774 ± 0.0018 |
| TLS-2-P | 409347 | 457 | 162.2 | 2.04 | 1.26 | 0.5247 ± 0.0020 |
| HX-P | 406654 | 726 | 688.0 | 1.02 | 0.15 | 0.3844 ± 0.0014 |
| WLXP-P | 404848 | 303 | 218.0 | 1.71 | 0.79 | 0.4536 ± 0.0017 |
| YY-P | 404846 | 295 | 230.6 | 0.64 | 0.28 | 0.4882 ± 0.0024 |
| YC-P | 404844 | 133 | 122.7 | 1.34 | 1.09 | 0.5163 ± 0.0019 |
